# Emergency department visits in older patients: a population-based survey

**DOI:** 10.1186/s12873-019-0236-3

**Published:** 2019-02-27

**Authors:** Mika Ukkonen, Esa Jämsen, Rainer Zeitlin, Satu-Liisa Pauniaho

**Affiliations:** 1 Department of Gastroenterology and Alimentary Tract Surgery, Tampere University Hospital, Tampere, Finland; 2 Emergency Division of Pirkanmaa Hospital District, Tampere University Hospital, Teiskontie 35, 33521 Tampere, Finland; 3 Centre of Geriatrics, Tampere University Hospital, Tampere, Finland; 4 Faculty of Medicine and Health Technology, Tampere University, Tampere, Finland; 5 Department of General Administration, Tampere University Hospital, Tampere, Finland

**Keywords:** Epidemiology, Emergencies, Aged, Health care costs

## Abstract

**Background:**

Given the higher incidence of emergency conditions in older inhabitants, the global increase in aged population will pose a challenge for emergency services. In this study we examined the burden caused to emergency health care by the aged population.

**Methods:**

Consecutive patients aged 80 years or over visiting a high-volume, collaborative emergency department (ED) between 2015 and 2016 were included. The key factors under analysis were the incidence of emergency conditions and costs associated with emergency care.

**Results:**

A total of 6944 patients (median age 85 years, range 80–104 years; 67% female) aged ≥80 years representing 1.5% of the local population, made 17,769 ED visits during the two-year observation period accounting for 15% of all ED visits. Forty-two percent (*n* = 2884) of patients had a single ED visit, whereas 8.2% (*n* = 570) made ≥5 ED visits/year for a total of 1400 visits (7.9%). Thirty-two percent of those aged ≥80 years required ED services each year. The number of ED visits increased with age (*p* < 0.001); and was 768/1000 person-years among octogenarians and 1007/1000 among nonagenarians, in comparison to 233/1000 among those aged < 80 years. One in five of the study population were discharged with non-specific diagnoses. Typical diagnoses included pneumonia (4.8%), malaise and fatigue (4.5%) and heart failure (4.3%). Non-specific diagnoses were frequent, and examination of patients with non-specific diagnoses incurred costs similar to or higher than those of other patients. The mean cost per ED visit in older patients was 422 €.

**Conclusions:**

We demonstrated a high incidence of emergency department visits in older patients. While our aim was not to solve how the growing demand should be met, it seems unlikely that increasing ED resources is feasible. Instead, the focus should be on chronic care of the aged and prevention of potentially avoidable ED visits.

## Background

The world population is ageing rapidly; from 2015 to 2050 the number of persons worldwide aged 80 and over will more than triple from 125 million to 434 million [1]. This demographic change is already under way in developed countries such as Finland, where the proportion of people aged 80 years and over has risen to 5.2% and will reach 12% before 2065 [2]. Furthermore, the current share of older inhabitants in Finland corresponds with global projections for 2050–2060 [1, 2]. Given the high incidence of emergencies in older inhabitants [3], older people may account for an increasing share of emergency department (ED) visits in the coming years.

Previous reports have already shown an increasing demand for ED services. For example in England the number of ED visits by patients aged 65 years or over increased 46% between 2001 and 2012 [4]. According to the National Centre for Health Statistics, in the USA the annual visit rate among persons aged 65 years or more was 511/1000 persons and increased with age [5]. Older patients have higher rate of hospitalization, require more resources and are at increased risk of adverse outcome [3, 5]. Consequently, the costs of care are higher than among those in younger cohorts.

For the reasons mentioned above, the primary aim of this study was to assess the burden on ED services caused by population ageing in Finland. The secondary aim in this population-based observational study was to assess the associated overall costs of ED care. As far as we know, no similar analyses have been previously reported.

## Methods

Consecutive older patients visiting a high-volume, collaborative ED during a two-year study period (January 2015 to December 2016) were included. The study population (i.e. older inhabitants) was defined as those being aged 80 years or over. In the study ED, all patients visit the same ED regardless of their indication for emergency visit and all ED physicians have similar options available to examine patients, i.e. the same radiological services and laboratory examinations are available to all physicians and for all patients. Specialists (i.e. internists, surgeons, neurologists and emergency physicians), residents and other acute care physicians work alongside each other.

The study hospital is a tertiary referral centre with catchment area exceeding one million inhabitants during the study period. The hospital ED also provides both primary and specialist ED care 24/7 within the city of Tampere with a population of 225,118 inhabitants (of which 10,852 were 80 years or over) in 2015 and 228,274 (11,129) in 2016. As we were interested specifically in including all emergencies, the city of Tampere was used for our study purposes. Only appointments with ED physicians were included.

The key factors under analysis were the incidence of ED visits and the associated cost of ED care. Finally, we attempted to assess the future need for ED services among older inhabitants using our incidence data and the population estimates provided by Statistics Finland.

### Incidence of ED visits

Both age- and gender-specific incidence rates (number of ED visits per 1000 same aged and same gender inhabitants) were calculated. Patient-related data was retrieved from hospital medical records. Population statistics were acquired from an open database provided by Statistics Finland (http://pxnet2.stat.fi/). Furthermore, specific subgroups, i.e. those making multiple ED visits were assessed separately. To measure the number of ED visits, each patient’s last ED visit was noted during the study period. This was referred to as the index ED visit. The number of previous ED visits was then counted for one calendar year. In this study we considered those requiring five or more visits to be high-frequency ED service users.

### Cost analysis

The cost analysis is based on the system in use in our hospital, which grades patients according to variables such as the time of day during the ED visit (i.e. higher costs during nights and weekends) and need for laboratory and radiological examinations. Consequently, the actual costs of individual examinations (i.e. the costs for each examination) could not be counted. Instead, the calculated cost represents how much the community (i.e. local taxpayers) is charged for the care. In the Finnish health care system, the majority of the costs are covered by taxes. The patients are charged a nominal fee of 32.70 € / ED visit regardless of the actual expenses. This fee paid by the patient was not taken into account in our cost analyses. Only in-hospital tax-funded costs of ED visit were included; costs of patient transfer, hospitalization and post-discharge care were not analysed. The tax-covered costs (€) per ED visit are presented. In the cost analysis, costs are also adjusted to the size of the same-aged population, and per capita costs (€ / same aged inhabitant) are then reported.

### Future of ED services

Finally, we provide estimates for the future development of the need for ED services among older inhabitants, which was done by projecting our age and gender specific incidence data (number of admissions per 1000 same-aged inhabitants) against population estimates provided by Statistics Finland (http://pxnet2.stat.fi/). Population projections provided by Statistics Finland are based on observations on past development in the birth rate, mortality and migration.

### Statistical analysis

All statistical analyses were performed using SPSS Statistics version 22 for Windows (IBM Corp, Armonk, NY, USA). Chi-square tests were performed to compare categorical variables and Student’s t-test continuous variables. Statistical significance was set at a *p*-value less than 0.05.

## Results

A total of 6944 patients (median age 85 years, range 80–104 years; 67% female) aged 80 years or over representing 1.5% of the local population, made 17,769 ED visits during the two-year observation period, accounting for 15% of all ED visits (*n* = 118,076). Forty-two percent (*n* = 2884) of older patients made a single ED visit, whereas 8.2% (*n* = 570) made 5 or more visits per year (range 5–46) during the two-year observation period, for a total of 1400 visits (7.9% of the total number of visits made by older patients). In 74% of cases patients (*n* = 13,158) were triaged to emergency room physicians, whereas 26% (*n* = 4611) were triaged to other specialists or residents (i.e. surgeons, internists or neurologists). Thirty-one percent of patients (*n* = 5423) were admitted to hospital (0.2%, *n* = 33 to intensive care units), 38% (*n* = 6755) were discharged home (or residential care) and the rest 31% (*n* = 5591) to other health care facilities.

**Fig. 1:**
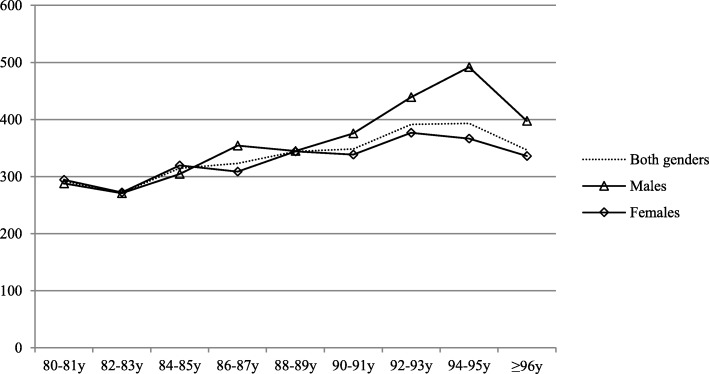
Population size adjusted number of those requiring ED care (number of patients admitted to ED/1000 same aged inhabitants/year) in different age (patients aged 80 years or over)

**Fig. 2:**
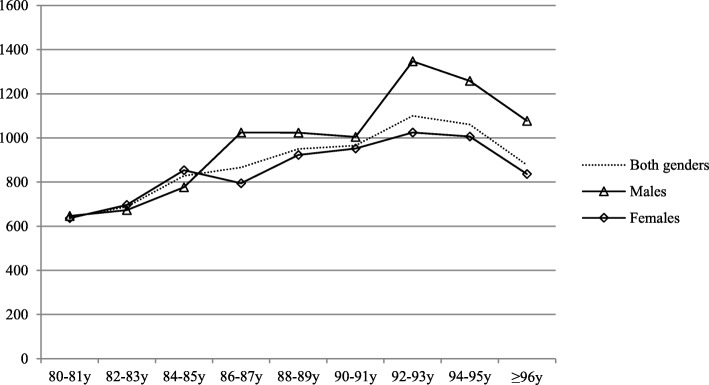
Age- and gender adjusted incidence of emergency department visits (total number of visits/1000 same aged inhabitants/year) among older inhabitants (aged 80 years or over)

Each year 32% of local population aged 80 years or over required ED services. The risk increased with age (*p* < 0.001); the corresponding rates among octogenarians (80–89 years) and nonagenarians (90 years or over) were 30 and 38%, as also shown in Fig. 1. The share of older inhabitants visiting ED was similar among males and females (32% vs. 31%, *p* = 0.590). The number of ED visits also showed an ascending trend with age; the number of ED visits per 1000 inhabitants among those aged 80–89 years and those over 90 years were 768 and 1007 respectively, while those aged less than 80 years had 233/1000/year ED visits. The number of ED visits in different age groups is shown in Fig. 2.

The reasons for an ED visits ranged from non-specific and mild symptoms to specific conditions with severe presentations. One in every five older patients (incidence 164/1000 person-years) was diagnosed with non-specific symptoms. When adjusted to the size of the same-aged population the incidence of non-specific diagnoses was higher among nonagenarians than among octogenarians (199/1000 vs. 157/1000, *p* < 0.001). Other typical diagnoses included pneumonia (4.8%, 38/1000), malaise and fatigue (4.5%, 36/1000), heart failure (4.3%, 35/1000), atrial fibrillation (4.1%, 33/1000) and non-specific abdominal pain (2.8%, 22/1000). Infections were common among the oldest patients, for example urinary tract-related infections were diagnosed in 9.5% (94/1000) of those aged 90 years or over. The distribution of typical diagnoses in different age groups is presented in Table 1.

**Table 1:** Distribution of typical principal diagnoses and complaints (ICD-10) in older patients (aged≥80 years) admitted to a collaborative emergency department

Diagnosis (ICD-10)	Octogenarians *n* = 14,061		Nonagenarians *n* = 3708		All patients *n* = 17,769	
Pneumonia (J18)	4.7%	1.	5.2%	3.	4.8%	1.
Malaise and fatigue (R53)	4.3%	2.	5.3%	2.	4.5%	2.
Heart failure (I50)	4.0%	4.	5.4%	1.	4.3%	3.
Atrial fibrillation (I48)	4.5%	3.	2.9%	7.	4.1%	4.
Cystitis (N30)	2.5%	7.	3.4%	4.	2.7%	5.
Head wound (S01)	2.4%	9.	3.3%	5.	2.6%	6.
Urinary tract infection, site not specified (N39)	2.4%	10.	3.2%	6.	2.5%	7.
Non-specific chest pain (R07)	2.5%	8.	2.3%		2.5%	8.
Unspecified disorientation (R41)	2.7%	6.	1.6%		2.4%	9.
Non-specific abdominal pain (R10)	2.9%	5.	2.4%		2.4%	10.
Intracranial injury (S06)	2.1%		2.9%	9.	2.3%	
Pyelonephritis (N10)	2.1%		2.9%	9.	2.3%	
Back pain (M54)	2.3%		2.5%		2.2%	
Hip fracture (S72)	1.7%		2.9%	8.	1.9%	

The most common diagnoses in hospitalized patients included hip fracture (6.2%), atrial fibrillation (5.4%) and pneumonia (5.3%), while among patients discharged home the most common diagnoses were atrial fibrillation (4.4%), head wound (4.2%) and non-specific chest pain (3.9%). The comparison between hospitalized and discharged patients is shown in Table 2. When those with a single visit and multiple visits were compared, the most notable difference was noted in the risk of injury-related visits, which was lower among those requiring ED services multiple times (17% vs. 12%, *p* = 0.042). Among high-frequency ED service users (≥5 visits/year) the share of those with injury-related visits was low (1.6%). Typical diagnoses among high-frequency users were heart failure (16%), atrial fibrillation (13%), pneumonia (12%), non-specific abdominal pain (10%) and urinary retention (8.6%). The comparison between those making a single visit and multiple visits is presented in Table 3.

**Table 2:** Comparison between those requiring hospital care in tertiary care services and those discharged home

Variable	Hospitalized ^a^ *n* = 5423	Discharged ^b^ *n* = 6755	All patients *n* = 17,769
Gender, female, %	64.9%	68.9%	67.7%
Age, median (range)	85y (80-103y)	85y (80-104y)	85 (80-104y)
Octogenarians, %	80.1%	83.6%	79.1%
Nonagenarians, %	19.9%	16.4%	20.9%
Distribution of diagnoses ^c^, %			
Non-specific symptoms	17.0%	23.2%	20.3%
Cardiovascular diseases	18.7%	10.2%	13.4%
Injuries	11.5%	15.8%	12.9%
Genitourinary diseases	3.8%	5.8%	7.0%
Pulmonary diseases	6.2%	3.4%	6.4%
Musculoskeletal diseases	1.7%	7.3%	4.8%
Infectious diseases ^d^	3.0%	1.7%	3.1%
Gastrointestinal diseases	5.2%	2.6%	3.1%
Mental illnesses	1.4%	1.3%	1.7%
Nervous diseases	2.1%	1.2%	1.6%
Miscellaneous	29.4%	27.5%	25.7%

^a^ Hospitalized in our hospital’s tertiary care services

^b^ Discharged home or residential care

^c^ Distribution of diagnoses and complaints, according to ICD-10 classification

^d^ According to ICD-10 classification subclasses ‘A00-A99’, which does not include all infectious conditions, e.g. urinary tract related infections classified in subclass ‘N’

**Table 3:** Comparison between those with a single ED visit and multiple ED visits

Variable	Single visit *n* = 2884	Multiple visits		All visits *n* = 17,769
		≥2 visits *n* = 14,885	≥5 visits/year *n* = 1400	
Gender, female, %	66.8%	68.3%	66.3%	67.7%
Age, median (range)	85y (80-102y)	86y (80-104y)	86y (80-104y)	85 (80-104y)
Octogenarians, %	83.4%	78.3%	79.9%	79.1%
Nonagenarians, %	16.6%	21.7%	20.1%	20.9%
Distribution of diagnoses, %				
Non-specific symptoms	19.0%	20.6%	22.0%	20.3%
Cardiovascular diseases	14.3%	13.3%	14.4%	13.4%
Injuries	16.5%	12.2%	1.6%	12.9%
Genitourinary diseases	5.6%	7.3%	7.3%	7.0%
Pulmonary diseases	7.1%	6.3%	7.9%	6.4%
Musculoskeletal diseases	5.3%	4.7%	3.0%	4.8%
Infectious diseases ^a^	2.6%	3.2%	4.5%	3.1%
Gastrointestinal diseases	2.7%	3.1%	4.4%	3.1%
Mental diseases??	1.6%	1.7%	2.1%	1.7%
Nervous diseases	2.1%	1.5%	2.2%	1.6%
Miscellaneous	23.2%	26.1%	30.6%	25.7%

^a^ According to ICD-10 classification subclass ‘A00-A99’, which does not include all infectious conditions, e.g. urinary tract related infections classified in subclasses ‘N10, N30 and N39’

**Fig. 3:**
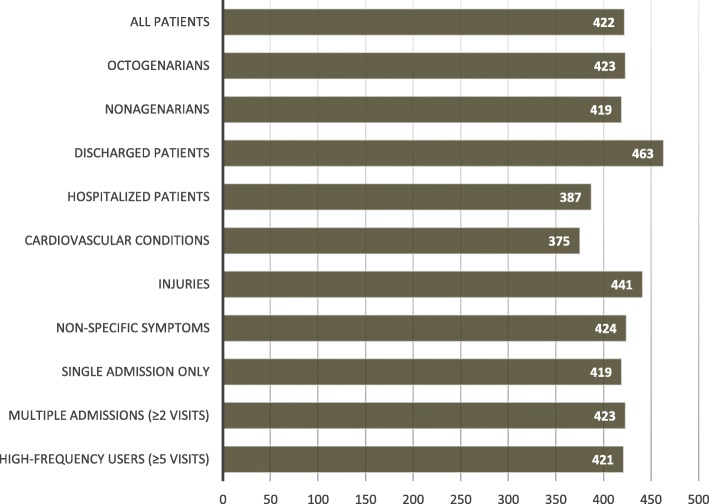
Mean public costs to the public (euros) per emergency department visits of older patients (aged≥80 years) in different patient populations

The mean cost per ED visit in older patients was 422 €. There was some difference in the costs depending on patient selection; for example, the mean costs of ED visits were higher among those discharged than among those requiring hospitalization (463 € vs. 387 €, *p* < 0.001), as illustrated in Fig. 3. When adjusted to the size of the population, there was a steady increase in the costs according to age; the costs (per capita) in patients aged 90 years or over were 1.3-fold compared to those of patients aged 80–89 years (420 € vs. 320 €, *p* < 0.001). Older patients often required resource-intensive examinations. Sixteen percent of patients (*n* = 2886) underwent CT scan and 3% (*n* = 536) ultrasonography.

**Fig. 4:**
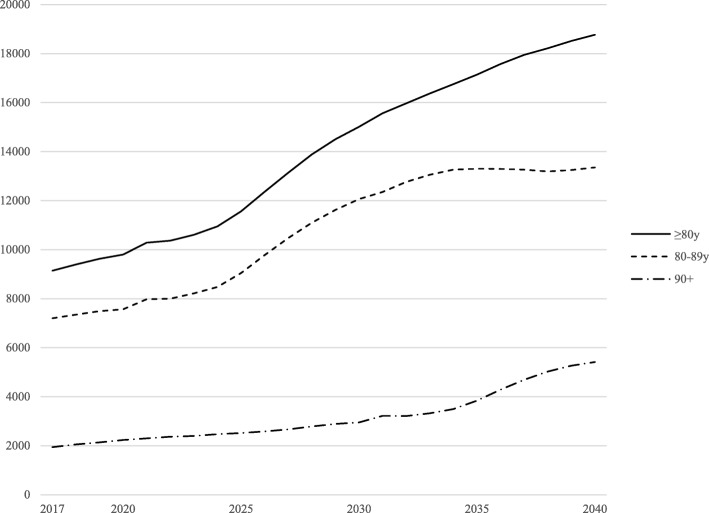
Estimated annual number of emergency visits within our hospital district based on age and gender-adjusted incidence (number of admissions per 1000 same-aged inhabitants) and population projections by Statistics Finland [2] (years 2017 to 2040)

When our incidence rates are compared against population estimates [2], there will be a rising trend in the need for ED services as the older population increases. The number of ED visits of those aged 80 years or over will more than double during the next 20 years, as shown in Fig. 4. The greatest increase will be observed among those aged 90 years or more. Similarly, applying current incidence rates to population estimates the number of visits among those aged less than 80 years will increase by only 9%.

## Discussion

In this study we report a high incidence of ED visits in older patients. The costs of ED care were considerable and did not differ significantly, for example, between those with non-specific diagnoses and those of patients requiring hospitalization for serious acute conditions. Thus, it seems that those with non-specific complaints often require resource intensive examinations to exclude the possibility of specific acute conditions.

We found an over three-fold increase in the incidence of emergency visits among those aged 80 years or over compared to that of those aged less than 80 years. Nearly one third of local inhabitants aged 80 years or over required ED services each year, and the total number of ED visits per 1000 same-aged inhabitants was over 1000 among those aged over 90 years compared to that of 233 among those aged less than 80 years. Given the wide variation in how emergency services are organized globally, population ageing is likely to have an impact on the rate of ED visits in the coming years [5–7]. Furthermore, in this study the costs of ED care were significant. Older patients often present with vague, intermittent and non-specific symptoms, which are often difficult to differentiate from specific and serious acute conditions. Consequently, those with non-specific symptoms undergo expensive examinations more often than those with specific acute conditions. These findings are in line with earlier studies showing that older patients frequently require more resources than do younger patients, outcome is poorer and these patients require hospitalization more often [3, 5].

Based on our findings we assumed that the number of ED visits in our study population will more than double in the next 20 years. Similar ascending trend in ED use by older patients may be observed globally as the proportion of older patients and the projected population demographics in our particular region mimic those in most parts of western Europe, the USA and the UK, for example [1, 2]. While a steady transition towards large and multidisciplinary emergency services is likely to improve the care of critically ill patients, it may not respond appropriately to the increasing needs of very old patients [3]. Recognizing common geriatric syndromes, like cognitive decline, malnutrition and falls, underlying the ED presentation [8] might help in cutting the need for different examinations in patients with non-specific symptoms although these patients are also at high risk of suffering from serious acute conditions [9]. Indeed, specialized geriatric teams may improve the emergency care of older patients [3, 10, 11]. On the other hand, the on-call availability of a geriatric specialist or emergency service may help to avoid unnecessary transfers for those for whom the potential adverse events of transfer are likely to outweigh the benefits of in-hospital care [12]. Until such services become available in our health care system, good care of patients’ chronic conditions and management of geriatric syndromes in order to maintain their physical condition and reduce the need for emergency care. In the lack of clinical details, we could not estimate what share of the ED visits could be prevented. Previously precautionary actions have demonstrated some impact on the incidence of emergencies, for example on hip fractures in older population [13].

This study has some limitations. First, we described ED service use in a single Finnish community. However, by focusing on this specific community we were able to access accurate information on all ED visits within the region. In addition, we were also able to obtain precise population statistics and pre-calculated demographic projections. By limiting the study population to those aged 80 years or over, we were intentionally focusing on very specific – likely frail – population. Furthermore, we have shown that ED visits among those aged less than 80 years were significantly less common than among those aged 80 years and over. Second, as our data were based on discharge records, we were unable to estimate the proportion of potentially avoidable ED visits. In some cases, the final diagnosis could not be set during the ED visit, which explains some of the visits with unspecific diagnoses. The most crucial limitation is that we were particularly interested in the in-hospital costs of ED care and examinations, and hence ignored the costs of hospitalization, for example. The costs of patient transfers and hospitalization certainly play an important role in the overall costs of care. Furthermore, our cost analysis cannot be representative of ED care globally. However, we emphasize that the economic pressure caused by the ageing population will be substantial, and should already be among the key motivators for improving the system before the economic pressure becomes intolerable.

## Conclusions

In our study there was a three-fold increase in ED visits made by patients 80 years or more compared to those under 80, and aging of the population will lead to a rapid increase in the number of ED visits. Non-specific diagnoses were frequent and generated similar or higher costs to those of patients requiring hospitalization. These observations indicate that we need a strategy to meet the growing demand for emergency services. Successful management of the acute concerns of older persons requires models of care that emphasize continuity and enabling of existing services. Many ED visits could be potentially prevented by optimizing the management of chronic conditions, easy and rapid access to other community-based primary care services and sufficient physician support for home care nurses. When acute care is needed, appropriate training for emergency physicians and nurses in dealing with geriatric issues might help in avoiding unnecessary examinations and improving prompt management of geriatric patients. However, the demographic and clinical characteristics of elderly ED patients need to be more comprehensively described in order to improve the risk assessment and identification of patients with specific, geriatric needs.

## Abbreviation

ED: Emergency department
